# Protein Chemical Labeling Using Biomimetic Radical Chemistry

**DOI:** 10.3390/molecules24213980

**Published:** 2019-11-03

**Authors:** Shinichi Sato, Hiroyuki Nakamura

**Affiliations:** Laboratory for Chemistry and Life Science, Institute of Innovative Research, Tokyo Institute of Technology, Yokohama 226-8503, Japan

**Keywords:** biomimetic radical reaction, bioinspired chemical catalysis, protein labeling

## Abstract

Chemical labeling of proteins with synthetic low-molecular-weight probes is an important technique in chemical biology. To achieve this, it is necessary to use chemical reactions that proceed rapidly under physiological conditions (i.e., aqueous solvent, pH, low concentration, and low temperature) so that protein denaturation does not occur. The radical reaction satisfies such demands of protein labeling, and protein labeling using the biomimetic radical reaction has recently attracted attention. The biomimetic radical reaction enables selective labeling of the C-terminus, tyrosine, and tryptophan, which is difficult to achieve with conventional electrophilic protein labeling. In addition, as the radical reaction proceeds selectively in close proximity to the catalyst, it can be applied to the analysis of protein–protein interactions. In this review, recent trends in protein labeling using biomimetic radical reactions are discussed.

## 1. Introduction

The development of a technique for covalent bond formation between a specific amino acid residue of a protein and a low-molecular-weight compound is an important issue in protein chemical labeling and the design of protein-based biomaterials. It is also indispensable for the development of antibody–drug conjugates (ADCs) that have attracted attention in recent years. In addition, a technique for selectively labeling a specific protein in a complex protein mixture is useful for the target identification of bioactive molecules. In order to achieve protein chemical labeling, it is essential to develop reactions that result in the formation of covalent bonds with natural proteins in water, at near-neutral pH, at temperatures below 37 °C, and within a short reaction time of a few hours. Methods for labeling nucleophilic amino acid residues (lysine and cysteine residues) using compounds with electrophilic properties have been developed and have greatly contributed to the advancement of biochemistry. Additionally, site-selective protein labeling techniques [1] and enzymatic protein labeling techniques have been developed in recent years [2]. On the other hand, the chemical modification of amino acid residues, other than lysine and cysteine residues, has been extensively studied in recent years. The selective modification of tyrosine residue [3,4,5,6,7,8,9,10,11,12], tryptophan residue [3,13,14,15,16,17,18], methionine residue [19,20], peptide chain N-terminus [21,22], and the C-terminus [23] can also be used for protein functionalization. Radical reactions can modify amino acid residues that cannot be modified by conventional electrophilic methods, or modify proteins/peptides with a novel binding mode (e.g., stable C–C bond formation). In this review, we focus on protein labeling reactions using the bioinspired single-electron transfer (SET) reaction.

## 2. Biomimetic Tyrosine Radical Labeling Using Enzymes

**Figure 1:**
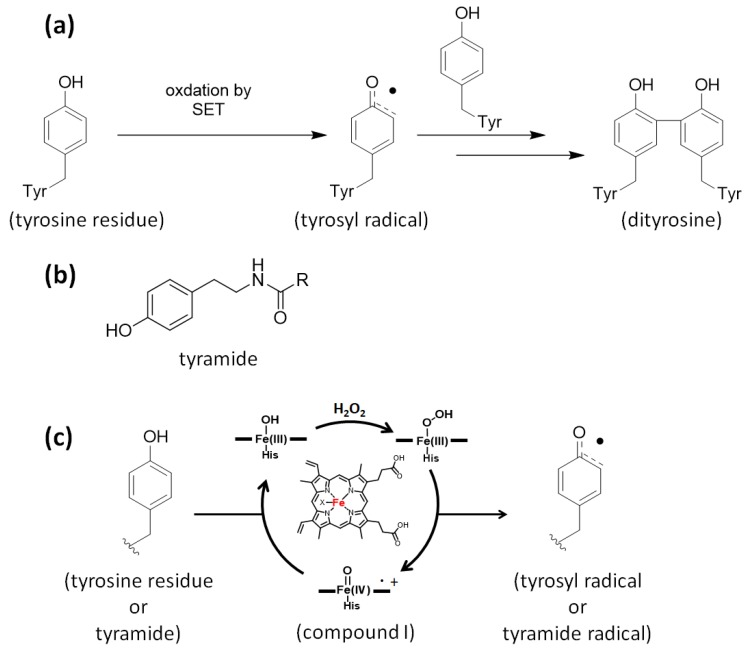
Generation of tyrosyl radical and tyramide radical. (**a**) Mechanism of dityrosine generation via single-electron transfer (SET). (**b**) Tyramide, a labeling agent that mimics tyrosine (**c**) Mechanism of oxidation in the active site of horseradish peroxidase (HRP).

In the biological radical reaction called radiolysis, water breaks down to highly reactive radicals such as hydroxyl radical, superoxide anion radical, and H_2_O_2_ [24]. Although the disulfide bond forming reaction is widely known as a response to oxidative stress in living systems, a dityrosine structure resulting from an oxidative cross-linking reaction of a tyrosine residue has also been reported as a protein oxidative modification marker [25,26]. Tyrosine readily undergoes SET under oxidative conditions to produce a highly reactive tyrosyl radical. A dityrosine structure is formed by the dimerization of tyrosine residues through the generation of tyrosyl radicals. Tyramide, a labeling agent that mimics tyrosine, forms a covalent bond with a tyrosine residue in a manner similar to dityrosine (Figure 1). Mimicking the biological response of dityrosine formation, metal complexes such as Ni(III) and Ru(III) were also reported to generate tyrosyl radicals and the radical species of tyramide. They were also used for protein cross-linking and protein labeling [27,28]. Several types of metalloenzymes, including peroxidase, tyrosinase [29,30,31], and laccase [32,33], catalyze the oxidation of tyrosine residues. As tyrosyl radical generation is efficiently catalyzed by peroxidases such as horseradish peroxidase (HRP), peroxidase was utilized as the catalyst in the dityrosine cross-linking reaction (Figure 1) [34,35,36,37,38,39,40]. HRP is activated by H_2_O_2_, and heme in the HRP molecule is transformed into a highly reactive species called compound I ([PPIX]·+Fe(IV)O), which can abstract a single electron from tyrosine or tyramide with ~1.1 V redox potential [41].

**Figure 2:**
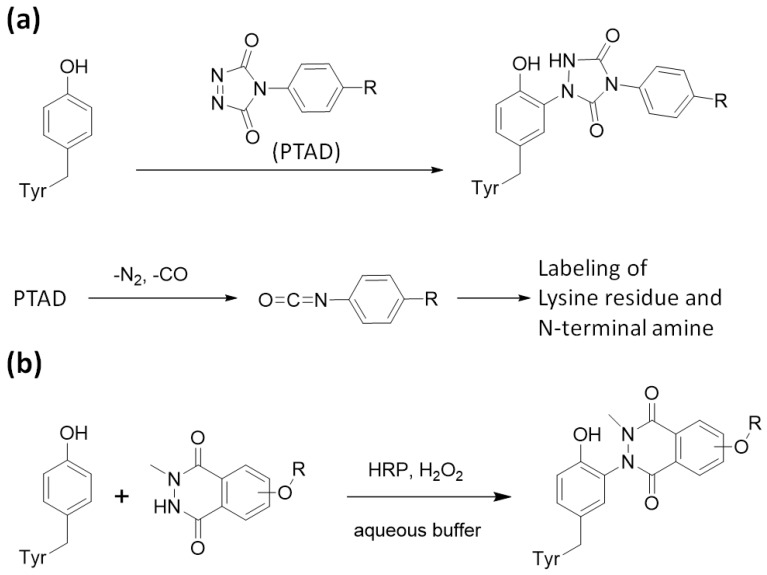
Tyrosine labeling with PTAD and *N*-methylated luminol derivatives. (**a**) Tyrosine labeling with PTAD and side reaction with amine group via isocyanate generation. (**b**) Tyrosine labeling with *N*-methylated luminol derivative in the presence of HRP and H_2_O_2_.

Aside from the tyrosine labeling reactions, other than mimicking dityrosine formation reaction, a tyrosine labeling reaction that uses 4-phenyl-1,2,4-triazoline-3,5-dione (PTAD) as the labeling agent was reported [10,42]. However, PTAD easily decomposes in water to form isocyanate, an active electrophile. Therefore, the resulting isocyanate reacts not only with tyrosine residues but also with electrophilic amino acid residues and the *N*-terminus. To achieve tyrosine-specific labeling, we developed tyrosine labeling agents based on the structure of luminol and found that tyrosine-specific labeling can be achieved under biomimetic radical oxidation conditions [43,44]. The idea originated from a reactive intermediate of the luminol chemiluminescence reaction, which has a cyclic diazodicarboxamide structure in common with PTAD. However, unlike PTAD, the luminol derivative selectively reacts with tyrosine residues without generating an electrophilic by-product. Various heme proteins and enzymes were tested as catalysts for oxidative tyrosine labeling reactions, and it was found that HRP effectively catalyzes the oxidative activation of luminol derivatives and induces tyrosine-specific modifications (Figure 2). Through the structure–activity relationship studies of luminol derivatives as tyrosine labeling agents, we revealed that *N*-methylated luminol derivatives labeled tyrosine residues efficiently, instead of showing chemiluminescent properties. The redox potential of activated HRP (~1.1 V) is sufficient to activate SET reactions between compound I (Figure 1) and *N*-methylated luminol derivatives, resulting in a radical activation labeling agent. Tyrosine residues in proteins and peptides were selectively and efficiently labeled with *N*-methylated luminol derivatives under HRP-activated conditions.

## 3. Peroxidase-Proximity Protein Labeling

**Figure 3:**
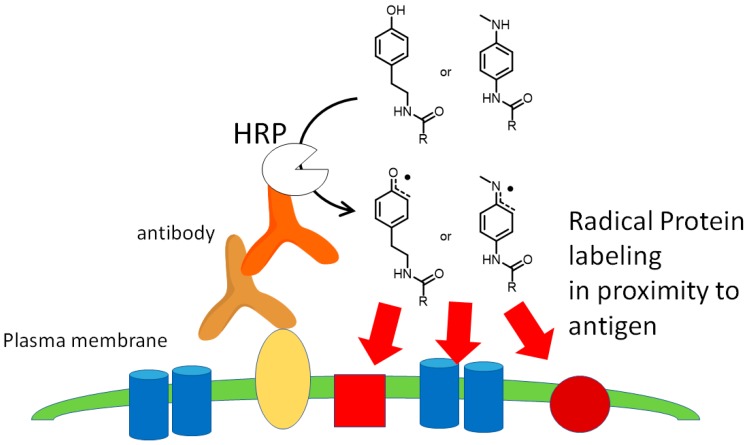
Immunohistochemical signal amplification using HRP-proximity protein labeling. Tyramide and *N*’-acyl-*N*-methylphenylenediamine were reported as HRP-proximity protein labeling agents.

Radical protein labeling using peroxidase has been employed in various applications in biological research. In general, the biomimetic radical reaction proceeds selectively in close proximity to the catalyst because of the short lifetime of the generated radical species. This concept is called proximity-dependent labeling (PDL). PDL catalyzed by HRP bound on the secondary antibody is also used as a signal amplification method (tyramide signal amplification—TSA) for immunostaining in biochemistry [45]. Although several signal amplification methods have been reported [46,47,48,49,50], TSA using HRP and tyramide derivatives is the most widely used. The generated tyramide radical reacts with amino acid residues such as tyrosine, tryptophan, histidine, and cysteine [51,52], in close proximity to HRP [53]. We found the novel signal amplification agent *N*’-acyl-*N*-methylphenylenediamine instead of tyramide, and revealed that it could be applied to signal amplification using HRP with comparable efficiency to tyramide (Figure 3) [54].

PDL has also been applied to the analysis of protein–protein interactions. Methods using HRP have been reported, including selective proteomic proximity labeling assay using tyramide (SPPLAT) [53] and enzyme-mediated activation of radical sources (EMARS) [55]. With SPPLAT, proteins on the cell membrane can be labeled with biotin-tyramide using HRP-conjugated antibodies or HRP-conjugated ligands (e.g., HRP–transferrin). Membrane proteins labeled by proximity labeling can be enriched by streptavidin beads capture. Enriched proteins are identified by MS/MS analysis. Li and co-workers labeled membrane proteins using the SPPLAT method targeting the B cell receptor (BCR) and succeeded in identifying not only known proteins that interact with BCR but also proteins whose interactions were unknown [53]. EMARS is a method that uses biotin-aryl azide as the labeling agent. HRP activates aryl azide to produce short-lived aryl nitrene. Nitrenes are known to react with various amino acid residues, such as tyrosine, tryptophan, lysine, threonine, isoleucine, and proline [56]. Honke and co-workers demonstrated that many kinds of receptor tyrosine kinases (RTKs) formed clusters with beta-integrin by a combination of the EMARS method and antibody array analysis [55].

**Figure 4:**
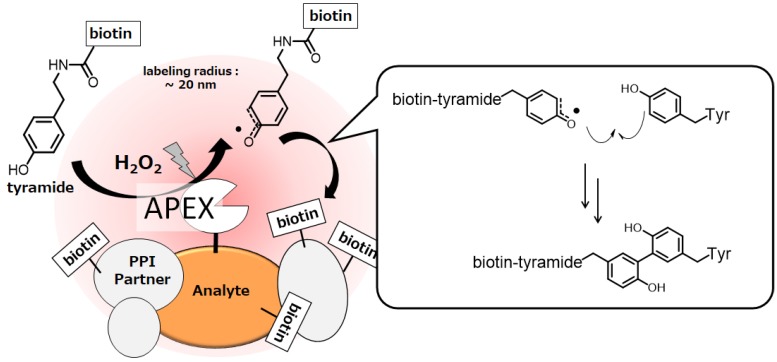
Ascorbate peroxidase (APEX) -proximity labeling of endogenous proteins in living cells.

The labeling radius from HRP by these methods ranges from less than 200 nm to 300 nm [53,55], which is suitable for analyzing protein clusters on cell membranes. However, HRP is inactive when expressed in mammalian cytosol. Considering that disulfide bonds and Ca^2+^ binding sites in the structure of HRP are not formed under intracellular reducing conditions and a Ca^2+^-scarce environment, Ting and co-workers focused on ascorbate peroxidase that lacks a disulfide bond and a Ca^2+^ binding site, and developed an engineered ascorbate peroxidase (APEX) that functions as peroxidase even in an intracellular environment [57]. In an intracellular environment, APEX catalyzes the generation of tyramide radical. The tyramide radical is short-lived (<1 ms) [58] and has a labeling radius of less than 20 nm [59,60] in the cells (Figure 4).

Ting and co-workers established a method for comprehensively labeling and identifying proteins expressed in specific organelles by fusing APEX to proteins expressed in specific organelles [52]. Furthermore, they developed APEX2, which showed much higher peroxidase activity than APEX among 10^6^ APEX mutants by the yeast-display evolution technique [61].

APEX2 has attracted much attention as a powerful tool for protein interaction analysis, and its applications include revealing proteomes in subcellular compartments [51,52,62,63,64,65], G-protein-coupled receptor complexes [66,67], subcellular transcriptome mapping [68,69], and APEX2-proximity RNA labeling [70,71].

## 4. Protein Labeling Using Photocatalyst

**Figure 5:**
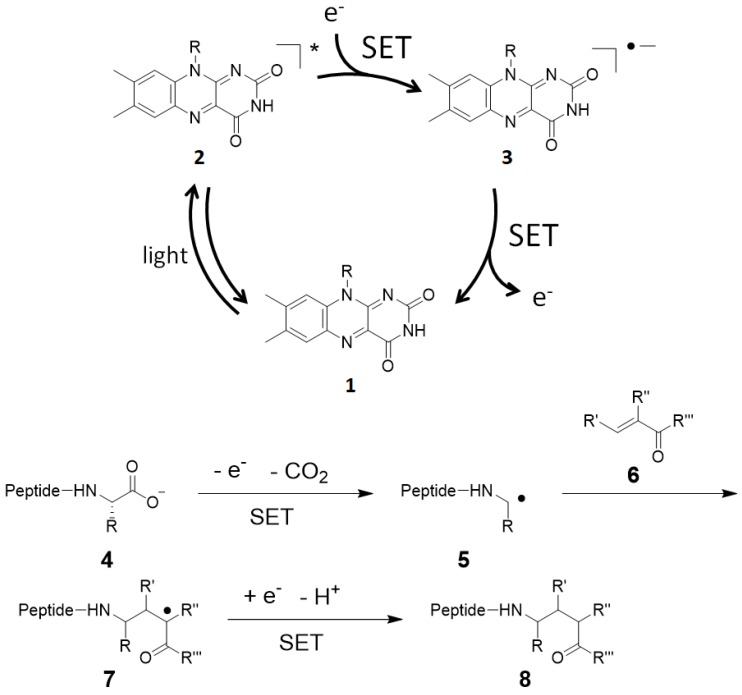
Proposed reaction mechanism for *C*-terminal labeling with flavin photocatalyst.

Not only radical enzymes but also small photocatalysts are used as protein labeling catalysts. Photocatalysts generate reactive oxygen species (ROS) and catalyze SET reactions in response to light stimulus [72]. Utilizing the SET mechanism, Noël and co-workers reported cysteine labeling using eosin Y and aryldiazonium salt [73], and Molander and co-workers reported a method that uses Ni/ruthenium photocatalyst and arylbromide [74]. MacMillan and co-workers developed a photocatalyst-mediated C-terminal labeling technique [23]. They focused on the redox potential of the carboxylic acid structures contained in the protein structure and hypothesized that the carboxyl group at the C-terminus would be selectively activated. The E_1/2_^red^ value of the carboxyl group in aspartic acid and glutamic acid residues is ~1.25 V (vs. saturated calomel electrode (SCE)), whereas the E_1/2_^red^ value of the C-terminal carboxyl group that exists at a single site in the protein sequence is ~0.95 V (vs. SCE). Slightly acidic reaction conditions (pH 3.5) are required in order to achieve efficient conversion, but the selective labeling of the C-terminus proceeded in the presence of aspartic acid and glutamic acid residues. MacMillan and co-workers tuned the reactivity of the Michael acceptor, a labeling agent, so that the labeling reaction with nucleophilic amino acid residues (lysine, serine, threonine, and histidine) would not proceed. The proposed reaction mechanism is shown in Figure 5. Flavin photocatalyst **1** is excited by visible light and undergoes subsequent intersystem crossing (quantum yield Φ_ISC_ = 0.38 for flavin in water at pH 7) and conversion into triplet-excited state **2**. Triplet-excited flavin is a strong single-electron oxidant (E_1/2_^red^ = 1.5 V vs. SCE in water) and should undergo facile SET with C-terminal carboxylate. Subsequent loss of CO_2_ from **4** furnishes nitrogen atom stabilized carbon-centered radical **5**. Radical **5** reacts with Michael acceptor **6** to produce carbonyl α-radical **7**. The photocatalyst in the radical anion state **3** reduces radical **7** to give product **8**, and regenerates ground-state photocatalyst **1**.

**Figure 6:**
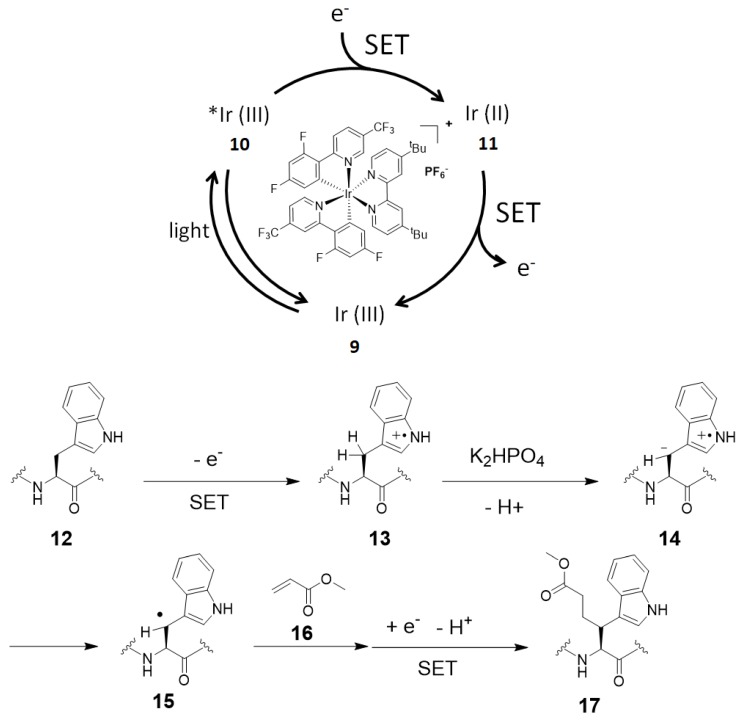
Proposed reaction mechanism for tryptophan β-position labeling with iridium photocatalyst.

Shi and co-workers reported a SET-mediated tryptophan modification at the β-position through C–H activation using Ir[dF(CF_3_)ppy]_2_(dtbbpy) complex as the photocatalyst [75]. They proposed a possible mechanism as shown in Figure 6. SET of the indole nitrogen atom generates radical cation **13**. The benzylic proton (β-position) of the tryptophan can be extracted by a base (K_2_HPO_4_) to form **14**, and subsequent electron transfer results to form more stable tryptophan radical **15**. The mechanism of generating N radicals by the dehydrogenation of indole NH from **13** can also be considered, but the β-position radical **15** contributes to the reaction. Radical **15** reacts with methyl acrylate **16** to generate another radical, and this is reduced by the iridium catalyst to afford labeled tryptophan product **17**. Although the Michael addition reactions with the amine group of lysine and the imidazole of histidine were also observed as side reactions, the modification proceeded selectively at the β-position of tryptophan and not at the β-position of tyrosine or phenylalanine in the reaction that used a peptide as the substrate.

**Figure 7:**
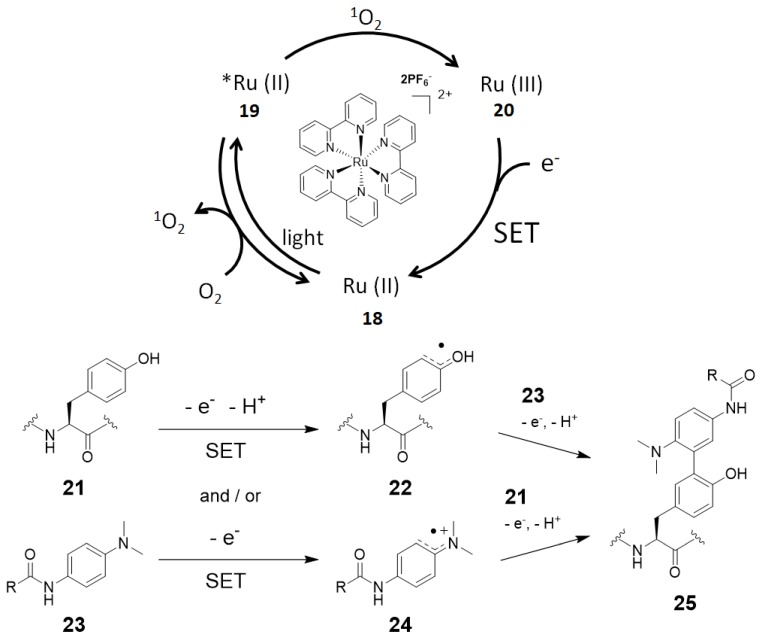
Proposed reaction mechanism for tyrosine labeling with ruthenium photocatalyst.

We also developed a tyrosine labeling method that uses Ru(bpy)_3_ complex and *N*’-acyl-*N*,*N*-dimethyl-1,4-phenylenediamine **23** as the photoredox catalyst and the labeling agent, respectively [11]. Under visible light irradiation, a stable carbon–carbon bond is formed between the ortho-carbon atom of the phenolic oxygen of the tyrosine residue and the ortho-carbon atom of the phenylenediamine derivative. Regarding the mechanism, in the absence of a labeling agent, ^1^O_2_ is generated by the catalyst that functions as a photosensitizer. ^1^O_2_ is involved in the production of Ru(III) active species **20**. Ru(III) active species **20** (1.1 V vs. SCE) can abstract a single electron from the tyrosine residue (~0.7 V vs. SCE) [76] and labeling agent **23** (0.63 V vs. SCE) [54]. Radical species **22** or **24** can react with **23** or **21**, respectively, to give product **25** through subsequent oxidation by SET (Figure 7) [77].

## 5. Photocatalyst-Proximity Labeling

**Figure 8:**
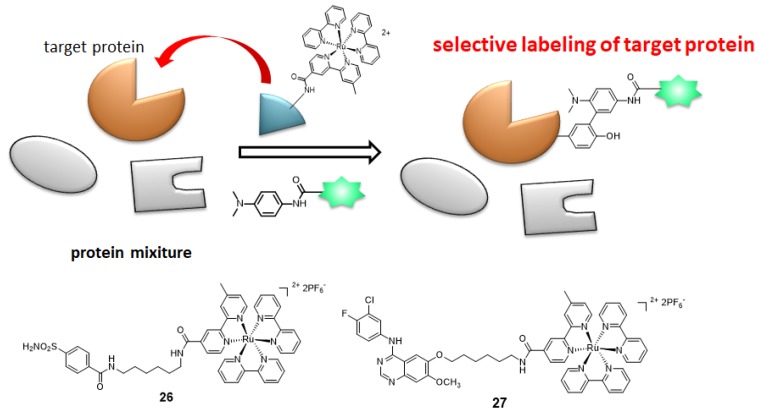
Target selective labeling by proximity labeling using ligand-conjugated photocatalysts **26** and **27**.

As mentioned in Section 3, photocatalyst-catalyzed radical protein labeling proceeds selectively in close proximity to a catalyst. Using this property, we designed a ligand-conjugated catalyst in which a ligand and a ruthenium catalyst were linked, and using this catalyst, we selectively labeled ligand-binding proteins in a protein mixture. As a proof-of-concept model, benzenesulfonamide-conjugated ruthenium complex **26** was synthesized for targeting carbonic anhydrase (CA). Mouse erythrocytes were incubated with **26** and photo-irradiated in the presence of the labeling agent. Despite the presence of various proteins in erythrocytes, CA was selectively labeled [11]. We also synthesized gefitinib-conjugated ruthenium catalyst **27**, which targets the epidermal growth factor receptor (EGFR) expressed in A431 cells, and succeeded in the selective labeling of EGFR in A431 cells [77]. Furthermore, we developed a method for target-selective purification and labeling using ruthenium-catalyst-functionalized affinity beads targeting CA and dihydrofolate reductase (DHFR) (Figure 8) [72].

**Figure 9:**
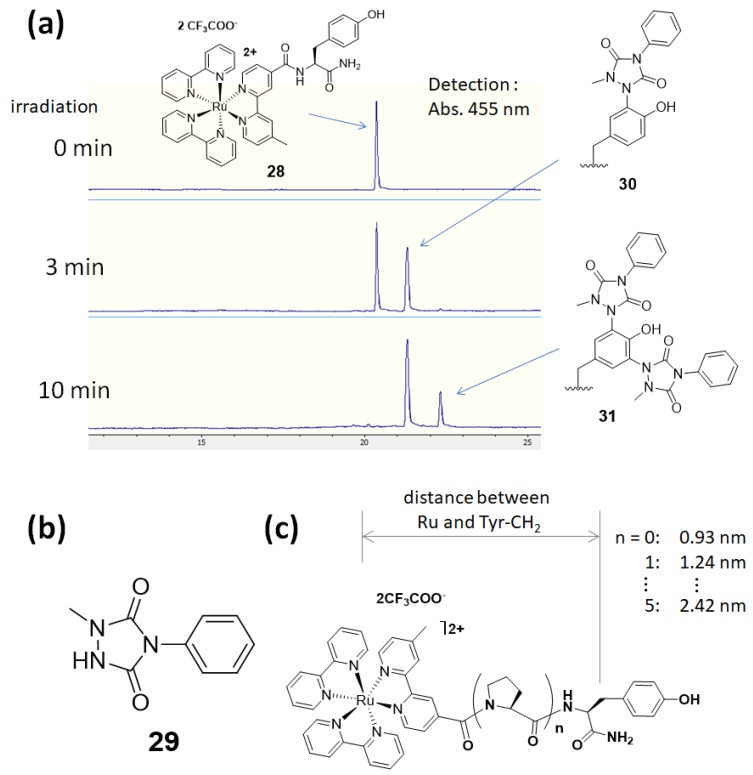
Photocatalyst-proximity tyrosine labeling. (**a**) Model substrate **28** was labeled with **29**. (**b**) Structure of labeling agent MAUra **29**. (**c**) Model substrate with a rigid proline linker with a distance of several nanometers between ruthenium and tyrosine.

**Figure 10:**
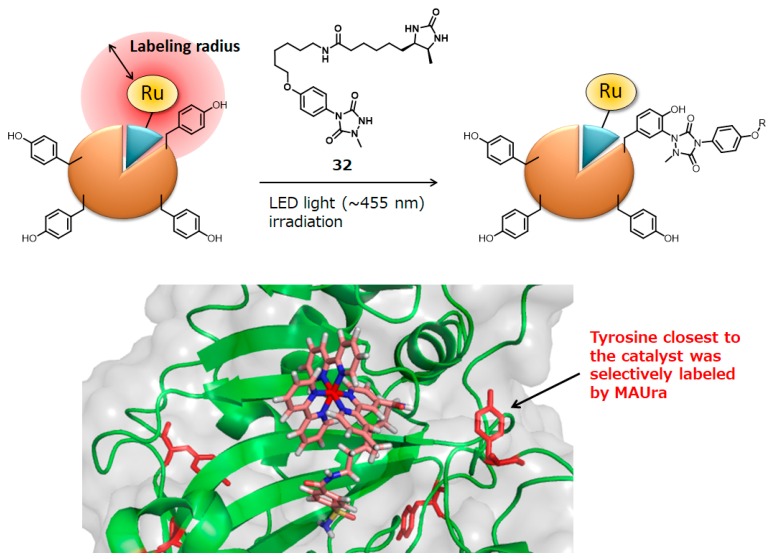
Photocatalyst-proximity labeling with MAUra.

In these applications, protein labeling in close proximity to the ruthenium photocatalyst was accomplished using *N*’-acyl-*N*,*N*-dimethyl-1,4-phenylenediamine **23** as the labeling agent. We also found a novel labeling agent that labels efficiently and selectively in nanometer-scale catalyst proximity. Using model substrate **28**, in which a tyrosine residue is linked to a ruthenium photocatalyst, the reaction efficiencies of various labeling agents were evaluated. It was found by LC-MS analysis that **28** was efficiently labeled with 1-methyl-4-aryl-urazole (MAUra, **29**) and converted into **30** and **31** (Figure 9). Furthermore, in order to estimate the labeling radius from the ruthenium complex, a ruthenium complex conjugated to tyrosine was synthesized with a rigid proline linker, in which the distance between ruthenium and tyrosine is several nanometers, as shown in Figure 9. MAUra (**29**) labeled tyrosine when a ruthenium complex and a tyrosine residue were in close proximity, and its distance dependence is not contradicted by the reported SET distance in a physiological environment (~1.4 nm) [78]. Desthiobiotin-conjugated MAUra **32** was used to selectively label CA in a protein mixture. The CA labeled with **32** was also successfully enriched using streptavidin beads (18.5% in two steps of labeling and enrichment). Identification of the labeling site by MS revealed that the tyrosine residue closest to the ligand binding site was selectively labeled, suggesting nanometer-scale proximity dependence of MAUra labeling (Figure 10) [12].

## 6. Electrochemical Protein Labeling

Protein modification using peroxidase or a photocatalyst is suitable for analyzing protein association and protein–protein interactions. However, it is necessary to develop an appropriate protein labeling agent according to the oxidation potential of each catalyst. Moreover, these methods sometimes require the addition of an oxidant, which is often a cause for concern about the oxidative damage of proteins. In recent years, protein labeling methods using electrochemistry have been reported to overcome this disadvantage. At present, electrochemistry is limited to labeling purified proteins, but in the case of electrochemical organic chemistry, the voltage applied to the reaction system can be adjusted easily and the reaction proceeds efficiently even in an aqueous buffer. It can be used for the functionalization of proteins because of its high amino acid residue selectivity and low oxidative damage.

**Figure 11:**
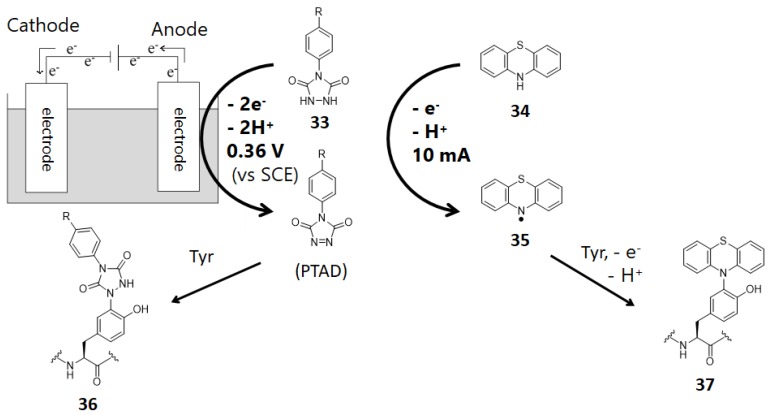
Electrochemical tyrosine labeling. SCE: saturated calomel electrode.

An electrochemical tyrosine-selective modification reaction (e–Y–Click) was reported by Alvarez-Dorta, Boujtita, Gouin, and co-workers (Figure 11) [79]. In this method, phenylurazole **33** is electrochemically oxidized and PTAD (Figure 2 and Figure 11) is gradually produced in the reaction system. Because the PTAD generated by anode oxidation reacts with tyrosine instantaneously, side reactions with nucleophilic residues and *N*-terminus via isocyanate formation can be suppressed. As phenylurazole undergoes anodic oxidation at 0.36 V (vs. SCE), peptides and proteins are labeled without severe oxidative damage. Using glucose oxidase (GOx) as the substrate, they confirmed that the enzymatic activity of GOx was not affected by tyrosine labeling through the e-Y-Click reaction. Lei and co-workers also reported tyrosine-selective electrochemical labeling using phenothiazine **34** as the labeling agent. (Figure 11) [80]. 

**Figure 12:**
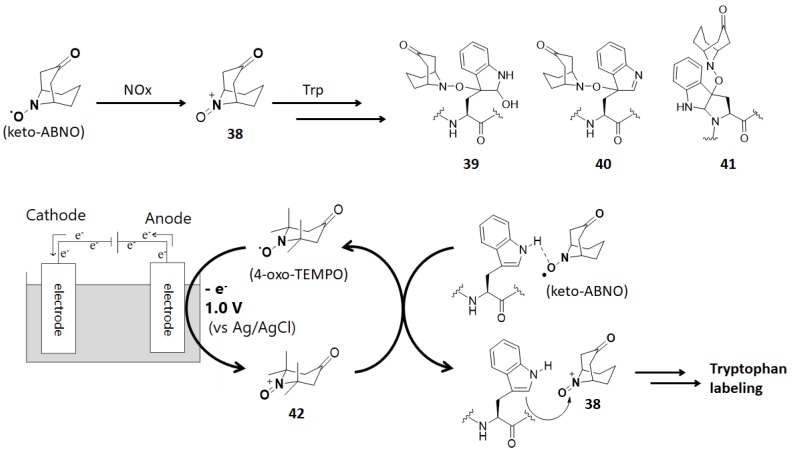
Tryptophan labeling with keto-ABNO and the electrochemical activation of tryptophan labeling.

Kanai, Oisaki, and co-workers reported that 9-azabicyclo [3.3.1]nonane-3-one-*N*-oxyl (keto-ABNO, Figure 12) selectively labels tryptophan residues in the presence of 0.1% acetic acid and NaNO_2_ [18]. Although keto-ABNO is oxidized by NOx in this method, they recently reported a method for activating the reaction by electrochemical oxidation [81]. They added 4-oxo-TEMPO as the electrochemical mediator to suppress both the anodic overoxidation of proteins and the cross reactivity to other amino acid residues (Figure 12).

## 7. Conclusions

In this review, protein labeling methods using biomimetic radical reactions were reviewed. Protein labeling techniques using electrophilic agents have been extensively employed. However, protein labeling targeting other amino acid residues is a challenging and attractive research topic. In recent years, in order to resolve several related issues, protein labeling using radical reactions has been actively developed targeting tyrosine and tryptophan residues and the C-terminus. Enzymes, particularly peroxidase, have been utilized as the catalyst for radical protein labeling, and peroxidase-proximity labeling has recently been used as an analytical method for protein association, protein–protein interaction, and transcriptome. In addition, protein modification using photocatalysts has been developed for the target identification of bioactive small molecules, and it is expected in the future to be used in not only the selective modification of target proteins in protein-mixed systems but also proximity labeling in cells. Furthermore, labeling with an electrochemical technique for precise voltage control has recently been developed and will be useful for labeling functional proteins. Table 1 summarizes representative protein labeling methods using biomimetic radical reactions. Future developments in radical protein modification will contribute to research on the elucidation of biological phenomena and drug delivery systems, and protein labeling using radical reactions will be a breakthrough technique in the development of these research areas.

## Figures and Tables

**Table 1:** Overview of protein labeling methods using biomimetic radical reactions.

Strategy		Labeling Agent	Target	Advantage	Disadvantage
**Enzyme**	Peroxidase	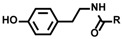 (tyramide)	Tyr (Trp, His, Cys)	Various biological applications Proximity labeling (see Section 3)	Use of H_2_O_2_ (1 mM ) Low efficiency
	Peroxidase	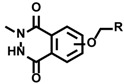 (N-Me luminol)	Tyr	High conversion Tyr selectivity	Use of H_2_O_2_ (~ 5 equiv.)
**Photocatalyst**	flavin	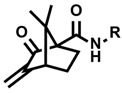 etc. (Michael acceptor)	*C*-terminus	Site-selective labeling	Low pH condition
	Ir[dF(CF_3_)ppy]_2_(dtbbpy)	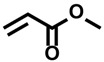 etc. (Michael acceptor)	Trp	β-position labeling Stable C-C bond	Cross reaction (with Lys, His)
	Ru(bpy)_3_	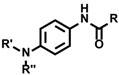 (phenylenediamine)	Tyr	Stable C-C bond Application to signal amplification (see Section 3)	Low membrane permeability of Ru catalyst
	Ru(bpy)_3_	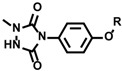 (MAUra)	Tyr	High efficiency Proximity labeling	Low membrane permeability of Ru catalyst
**Electrochemical**	0.36 V (vs. SCE)	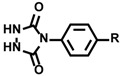 (phenylurazole)	Tyr	Mild condition	(All electrochemical methods) Not applicable to intracellular condition)
	10 mA	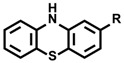 (phenothiazine)	Tyr	Tyrosine selectivity	Need > 50% CH_3_CN
	~ 1.0 V (vs. Ag/AgCl)	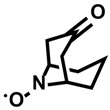 (keto-ABNO)	Trp	Tryptophan selectivity	Need high voltage
